# Determinants of Anti-S Immune Response at 12 Months after SARS-CoV-2 Vaccination in a Multicentric European Cohort of Healthcare Workers—ORCHESTRA Project

**DOI:** 10.3390/vaccines11101527

**Published:** 2023-09-26

**Authors:** Ludovica Leomanni, Giulia Collatuzzo, Emanuele Sansone, Emma Sala, Giuseppe De Palma, Stefano Porru, Gianluca Spiteri, Maria Grazia Lourdes Monaco, Daniela Basso, Sofia Pavanello, Maria Luisa Scapellato, Francesca Larese Filon, Luca Cegolon, Marcella Mauro, Vittorio Lodi, Tiziana Lazzarotto, Ivan Noreña, Christina Reinkemeyer, Le Thi Thu Giang, Eleonóra Fabiánová, Jozef Strhársky, Marco Dell’Omo, Nicola Murgia, Lucía A. Carrasco-Ribelles, Concepción Violán, Dana Mates, Agripina Rascu, Luigi Vimercati, Luigi De Maria, Shuffield S. Asafo, Giorgia Ditano, Mahsa Abedini, Paolo Boffetta

**Affiliations:** 1 Department of Medical and Surgical Sciences, University of Bologna, 40126 Bologna, Italy; ludovica.leomanni2@studio.unibo.it (L.L.);; 2 Department of Medical and Surgical Specialties, Radiological Sciences and Public Health, University of Brescia, 25121 Brescia, Italy; 3 Section of Occupational Medicine, Department of Diagnostics and Public Health, University of Verona, 37129 Verona, Italy; 4 Clinical Unit of Occupational Medicine, University Hospital of Verona, 37100 Verona, Italy; 5 Department of Medicine-DIMED, University of Padova, 35128 Padova, Italy; 6 Laboratory Medicine Unit, University Hospital of Padova, 35128 Padova, Italy; 7 Department of Cardiac Thoracic Vascular Sciences and Public Health, University of Padova, 35128 Padova, Italy; 8 Occupational Medicine Unit, University Hospital of Padova, 35128 Padova, Italy; 9 Occupational Medicine Unit, Department of Medicine, Surgery and Health Sciences, University of Trieste, 34100 Trieste, Italy; 10 SSD Health Surveillance, IRCCS University Hospital, 40139 Bologna, Italy; 11 Microbiology Unit, IRCCS University Hospital, 40139 Bologna, Italy; 12 Division of Infectious Diseases and Tropical Medicine, LMU University Hospital, 81377 Munich, Germany; 13 Department of Pediatrics, Dr. von Hauner Children’s Hospital, LMU University Hospital, 81377 Munich, Germany; 14 Occupational Health Department, Regional Authority of Public Health, 497556 Banská Bystrica, Slovakia; 15 Medical Microbiology Department, Regional Authority of Public Health, 497556 Banská Bystrica, Slovakia; 16 Unit of Occupational Medicine, Department on Medicine and Surgery, University of Perugia, 06125 Perugia, Italy; 17 Department of Environmental and Prevention Sciences, University of Ferrara, 44121 Ferrara, Italy; 18 Unitat de Suport a la Recerca Metropolitana Nord, Institut Universitari d’Investigació en Atenció Primària Jordi Gol (IDIAP Jordi Gol), 08303 Mataró, Spain; 19 Direcció d’Atenció Primària Metropolitana Nord Institut Català de Salut, 08007 Barcelona, Spain; 20 Grup de Recerca en Impacte de les Malalties Cròniques i les seves Trajectòries (GRIMTra), Institut Universitari d’Investigació en Atenció Primària Jordi Gol (IDIAPJGol), 08303 Barcelona, Spain; 21 Network for Research on Chronicity, Primary Care and Health Promotion (RICAPPS), Instituto de Salud Carlos III, 28029 Madrid, Spain; 22 Germans Trias i Pujol Research Institute (IGTP), 08916 Badalona, Spain; 23 Universitat Autónoma de Barcelona, 08193 Bellaterra, Spain; 24 National Institute of Public Health, 050463 Bucharest, Romania; 25 Department of Internal Medicine-Occupational Medicine, Medicine and Pharmacy “Carol Davila”, 050474 Bucharest, Romania; 26 Interdisciplinary Department of Medicine, University of Bari, 70121 Bari, Italy; 27 Stony Brook Cancer Center, Stony Brook University, Stony Brook, NY 11794, USA; 28 Department of Family, Population and Preventive Medicine, Renaissance School of Medicine, Stony Brook University, Stony Brook, NY 11794, USA

**Keywords:** COVID-19, coronavirus, vaccine, serology, antibody level, immunization, occupational health

## Abstract

Background: The effectiveness of the immunity provided by SARS-CoV-2 vaccines is an important public health issue. We analyzed the determinants of 12-month serology in a multicenter European cohort of vaccinated healthcare workers (HCW). Methods: We analyzed the sociodemographic characteristics and levels of anti-SARS-CoV-2 spike antibodies (IgG) in a cohort of 16,101 vaccinated HCW from eleven centers in Germany, Italy, Romania, Slovakia and Spain. Considering the skewness of the distribution, the serological levels were transformed using log or cubic standardization and normalized by dividing them by center-specific standard errors. We fitted center-specific multivariate regression models to estimate the cohort-specific relative risks (RR) of an increase of one standard deviation of log or cubic antibody level and the corresponding 95% confidence interval (CI) for different factors and combined them in random-effects meta-analyses. Results: We included 16,101 HCW in the analysis. A high antibody level was positively associated with age (RR = 1.04, 95% CI = 1.00–1.08 per 10-year increase), previous infection (RR = 1.78, 95% CI 1.29–2.45) and use of Spikevax [Moderna] with combinations compared to Comirnaty [BioNTech/Pfizer] (RR = 1.07, 95% CI 0.97–1.19) and was negatively associated with the time since last vaccine (RR = 0.94, 95% CI 0.91–0.98 per 30-day increase). Conclusions: These results provide insight about vaccine-induced immunity to SARS-CoV-2, an analysis of its determinants and quantification of the antibody decay trend with time since vaccination.

## 1. Introduction

Since the early phases of the SARS-CoV-2 pandemic, vaccines appeared necessary to contain the spread of infection and to prevent its consequences at the individual and community levels. The effectiveness of the new vaccines developed to confront the SARS-CoV-2 pandemic, like those based on mRNA (Comirnaty [BioNTech/Pfizer] [1] and Spikevax [Moderna] [2]), has been largely investigated with several studies demonstrating their different range of efficacy towards different outcomes, such as the infection, its symptoms and related hospitalization [3] and mortality [4,5,6]. The immunization level conferred by vaccines has been studied to predict the risk of infection and reinfection and the duration of vaccine protection, which raised special interest considering the timing of the pandemic and the outbreak of new variants of the virus [1,2].

Health care workers (HCW) are connoted by an inherent biological risk, which includes the risk of contracting infections, and were, therefore, a particularly exposed population to SARS-CoV-2, especially at the beginning of the pandemic when the appropriate use of personal protective equipment was not widespread [7].

HCW have been highly studied in relation to SARS-CoV-2 infection [8,9,10]. Being they were one of the first subgroups to be targeted with the new vaccines, one of the most exposed to the risk of infection [11] and continuously monitored at the occupational level during the COVID-19 pandemic [12], HCW represent a suitable population to investigate vaccine response.

In order to detect the humoral response to SARS-CoV-2, several tests are available. Anti-S1 levels (anti-Spike; Euroimmun, ELISA, anti-S1) showed a good correlation with neutralizing antibodies, which are a better immunity marker than anti-NP (anti-Nucleocapsid; Abbott, ELISA, anti-NP) [13]. The half-lives of neutralizing antibodies as anti-S antibodies are longer than anti-NP antibodies. The duration analysis shows that antibody decay follows a bi-phasic trend with increased half-lives of antibodies after 6 months and antibody persistence up to 14 months. Anti-spike antibody levels have been related to a long-lasting response, which exerts a stronger protection against reinfection [14]. However, the interpretation of SARS-CoV-2 antibody responses remains challenging, considering the high heterogeneity among study populations in terms of sociodemographic and clinical characteristics [15].

A recent systematic review of randomized controlled trials of SARS-CoV-2 vaccine efficacy and observational studies of SARS-CoV-2 vaccine effectiveness published from 17 June 2021 to 2 December 2021 found that SARS-CoV-2 vaccine efficacy or effectiveness against severe disease remained high, although it did decrease somewhat 6 months after full vaccination [16]. A similar result was also observed in a previous analysis of the ORCHESTRA study [17].

Conversely, other studies showed a decrease in serological response over time from SARS-CoV-2 vaccination. For example, a recent review conducted in Wuhan patients, analyzing the immune response to the SARS-CoV-2 vaccine, showed the antibody level decays to 64% of the starting level after 9 months from infection [18].

Our previous analysis within the ORCHESTRA project, including cohorts from several European countries, showed a decay of anti-SARS-CoV-2 IgG titers at 6 and 9 months after the second dose of the vaccine among HCW, despite the persistence of immune memory [10,19].

This study extends the previous analysis of the ORCHESTRA project and aims to provide additional evidence on the anti-SARS-CoV-2 spike immune response to COVID-19 vaccines at approximately 12 months after the first dose in a large population of HCW and identify the determinants of the immune response by investigating the characteristics related to HCW and their vaccination status.

## 2. Methods

ORCHESTRA is a European, multicenter, prospective cohort that involves more than 60,000 HCW employed in several hospitals in different countries, which started their collaboration on 1 December 2020 and will continue until 30 November 2024 [19]. This analysis investigates serological results at 12 months after the first vaccination dose in HCW from one center in Germany (Munich), seven centers in Italy (Bari, Bologna, Brescia, Padova, Perugia, Trieste and Verona with Bari comprising two subcohorts, Bari-IRCCS and Bari-Policlinico), one center in Spain (Northern Barcelona) and multiple centers in Slovakia and Romania (the latter two treated as an individual cohort).

We used medical surveillance records, appropriate questionnaires or local or regional databases to collect data on sociodemographic characteristics, results of PCR testing and vaccination status, including the dates of vaccination and types of vaccine. The results on the level of anti-S antibodies were either collected from the medical records or generated through adequate testing. All cohorts included in the ORCHESTRA project have undergone extensive data harmonization [17].

The present study comprises 16,101 HCW with available quantitative serology results after 12 months (defined as more than 330 days) from the first dose administration. Due to the missing data on the predictors in the regression analyses, 196 HCW (1.2%) were excluded, leaving 15,905 subjects in the analysis.

The measurement of the serologic level of anti-S antibodies at 12 months, which was considered complete data, was the main outcome of this analysis. Different centers in different time periods used various methods of detection of antibody levels, and the details are reported in Table 1.

In four cohorts, Bologna, Brescia, Perugia and Slovakia, between 70% and 92% of the results were above the cut-off value detected with the available assay. Therefore, we replaced, in these cohorts, the right-censored values with the predicted values obtained with a Tobit regression model, which used gender, age, previous SARS-CoV-2 infection and number of vaccine doses as predictors.

To address the skewness of the distributions, we applied a cubic transformation to the left-skewed data distribution (Bologna, Brescia, Perugia and Slovakia) and a log10 transformation to the remaining cohorts, which have right-skewed distributed data. We normalized the transformed values by dividing them by the cohort-specific standard deviation. The summary statistics of the serologic results are reported in Table 1.

We fitted multivariate linear regression models to estimate, for each cohort, the relative risks (RR) and corresponding 95% confidence intervals (CI) of an increase of one standard deviation (SD) of normalized antibody level. We considered several covariates: sex, age, time since last vaccine dose, previous SARS-CoV-2 infection, number of vaccine doses (one/two or three/four), type of vaccine (only Comirnaty [BioNTech/Pfizer]; Comirnaty [BioNTech/Pfizer] in combination with other vaccines except Spikevax [Moderna]; and Spikevax [Moderna] alone or in combination other vaccines) and job title (physician including resident, technician, nurse, administration and other HCW including auxiliary workers). Previous SARS-CoV-2 infection was assessed using either the results of anti-N antibody testing or based on the results of PCR. Cohort-specific results were combined using a random-effect meta-analysis.

The statistical analysis was conducted using the Stata^®^ software V. 17 (StataCorp LP, College Station, TX, USA) [20].

The pooled study was approved by the Italian Medicine Agency (AIFA) and the Ethics Committee of Italian National Institute of Infectious Diseases (INMI) Lazzaro Spallanzani. Each cohort was approved by the local ethical board.

## 3. Results

Overall, the analysis included 15,905 vaccinated HCW with complete data who provided blood samples 12 months after the first dose of the vaccine.

Table 2 reports selected characteristics of the study population stratified by cohort. Most subjects were from the Italy-Brescia (5569, 35%), Italy-Verona (2838, 18%), Italy-Padova (2336, 15%) and Italy-Trieste (2097, 13%) cohorts. The study population mainly consisted of women with the proportion ranging from 68% (Italy-Trieste) to 84% (Slovakia-Multicenter) and were aged more than 50 years (from 28% in Italy-Bari-IRCCS to 66% in Italy-Perugia). The most frequent job titles were nurse and physician in all the cohorts except for Slovakia-Multicenter and Italy-Perugia where the largest group was comprised of other HCW (including auxiliary workers). The majority of the population was never infected with SARS-CoV-2 (negative with PCR and/or anti N-antibodies) except in the Italy-Bari (Policlinico), Slovakia-Multicenter and Spain-Northern Barcelona cohorts where, respectively, 95%, 53% and 66% of the population examined had been infected at least once. Considering the type of vaccine administered, Comirnaty [BioNTech/Pfizer] was the most used in all the cohorts except Spain-Northern Barcelona where Spikevax [Moderna] was the predominant vaccine. In all the cohorts, the majority of the HCW received three or four vaccine doses, ranging between 58% (Slovakia-Multicenter) and 100% (Italy-Bari Policlinico). The mean of the standardized quantitative serology at 12 months varied between 2.00 (Italy-Perugia) and 15.54 (Spain-Northern Barcelona) after accounting for cubic transformation where appropriate. The mean of the lag time between the last dose of the vaccine and serology at 12 months varied from 49.86 in Italy-Verona (range: 10–350 days) to 226.13 in Slovakia-Multicenter (range: 20–424 days).

There was no difference in the serological response at 12 months according to the number of vaccine doses. Job title was not associated with serology level except for administration workers who showed an RR of 1.09 (95% CI = 1.02–1.16) compared to physicians. Finally, the subjects who were vaccinated with a combination of Comirnaty [BioNTech/Pfizer] and another vaccine, excluding Spikevax [Moderna], had a lower serological response compared to the subjects vaccinated only with Comirnaty [BioNTech/Pfizer] (RR 0.77; 95% CI = 0.60–0.98), whereas no difference was observed between Spikevax [Moderna] and Comirnaty [BioNTech/Pfizer] alone.

Supplementary Table S1 shows the results of the analysis stratified by the type of standardization. The results were broadly consistent between the two groups with the only differences being the lack of an effect of age and the presence of a stronger response following Spikevax [Moderna] vaccination in the cohorts with cubic transformation. The effects of the time since last vaccine dose and of previous SARS-CoV-2 infection were present in both groups of cohorts. The results of previous SARS-CoV-2 infection in relation to antibody level showed larger differences when considering PCR-based detection by timing of the infection in relation to vaccine administration with cubic standardization providing stronger associations than logarithmic standardization. Conversely, the anti-N serology test, which detected HCW who were ever infected, provided similar results, according to the two standardization methods.

**Figure 1:**
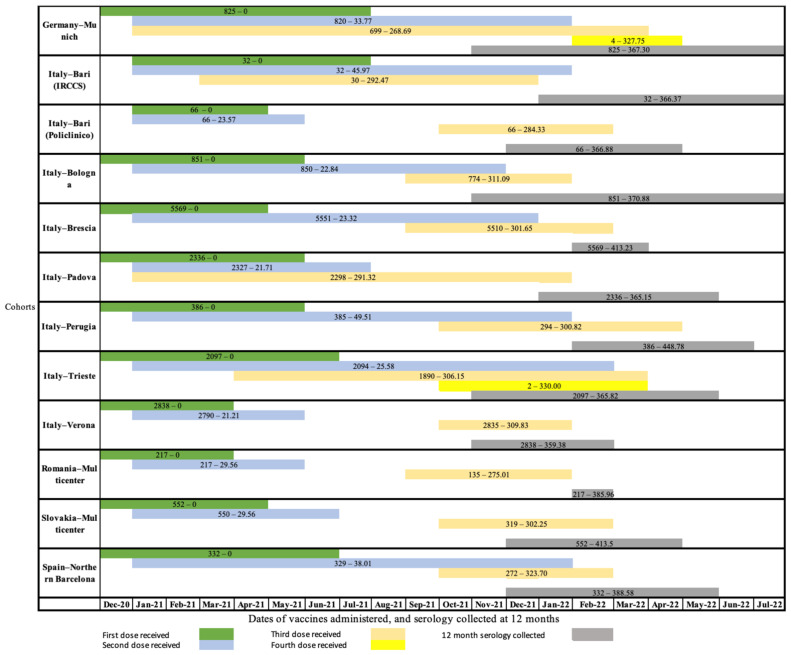
Timeline of serology collection and vaccine administration in each cohort. Notes: Number of subjects—mean of time since 1st dose of vaccine by cohort.

Figure 1 shows the timeline of serology sample collection and administration of vaccine doses in each cohort.

## 4. Discussion

The global impact of the COVID-19 pandemic has generated massive attention on the development of new vaccines to combat it, using different and sometimes new technologies: mRNA vaccines (like BNT16b2 Pfizer/BioNTech, mRNA-1273 Moderna and CVnCoV CureVac) consent the translation of viral antigenic proteins in vivo; viral vector vaccines (like AZD1222 ChAdOx1 nCoV-19 vaccine AstraZeneca/University of Oxford, Johnson and Johnson Ad26.COV2.S, Gam-COVID-Vac Sputnik V Gamaleya Research Institute) use viral vectors such as adenovirus to introduce into host cells viral genes that encode pathogen antigens; inactivated and protein subunit vaccines (such as CoronaVac Sinovac Biotech, NVX-CoV2373 Novavax, EpiVacCorona VECTOR) exploit the antigenic power of viral proteins to trigger protective immunity against it; and live attenuated virus vaccines use attenuated nonpathogenic virus immunogenicity to evoke host immunity [21,22]. Severe acute respiratory syndrome coronavirus 2 (SARS-CoV-2) contains three major structural proteins on the surface, spike (S), membrane (M) and envelope (E) proteins, while the nucleocapsid (N) protein binds viral RNA inside the virion. The M and E proteins have small molecular sizes and are poorly immunogenic. The N protein is highly immunogenic, but the first studies on the vaccines based on it showed that they do not confer protection. On the contrary, the S protein is the main target for the COVID-19 vaccines and consists of two subunits: S1, which includes the N-terminal domain (NTD) and the receptor-binding domain (RBD), and S2 [22].

The efficacy against the original strain of the virus and new emerging variants has been studied; after full immunization, mRNA vaccine effectiveness against the disease was 88–100% against Alpha, 76–100% against Beta/Gamma, 47.3–88% against Delta and 89–100% when the SARS-CoV-2 strain was not determined. AZD1222 efficacy against disease in the UK was 74.5% against Alpha and 67% against Delta. CoronaVac effectiveness was 36.8–73.8% against the Alpha/Gamma/D614G strain in Chile and Brazil. CoronaVac effectiveness in China was 59%. NVX-COV2373 had an efficacy of 89–91.6% against the historical strain, 86.3–93.2% against Alpha and 60% against Beta [21].

The great majority of our cohort was administered the mRNA vaccines, Comirnaty [Pfizer/BioNtech] and Spikevax [Moderna], also considering that HCW were between the first categories to benefit from the vaccinations, and the mRNA vaccines were the first to be approved for use.

This analysis addressed the SARS-CoV-2 anti-S levels in approximately 16,100 HCW from twelve different European cohorts collaborating in the ORCHESTRA project at 12 months from the first vaccination dose. Higher age, previous SARS-CoV-2 infection and use of the Spikevax [Moderna] vaccine were identified as predictors of higher anti-S serological levels. Moreover, antibody level was inversely related to the time from the first vaccination, supporting the progressive waning of this immunity marker.

In our previous analysis, female sex was associated with a higher antibody level [10,17], and similar findings have been reported in previous publications [23,24,25]. In this analysis, a higher antibody level was detected in men; albeit, the difference was not statistically significant. The correlation of immune response with sex is still an interesting field to be explored, as previous publications offered inconsistent results. For example, a recent systematic review did not find a difference in the efficacy of SARS-CoV-2 vaccines between men and women [26]. Moreover, an Asian study on the immunologic response after two doses of the Comirnaty [BioNTech/Pfizer] vaccine reported that, although the spike-reactive CD4+ T cell response was comparable between men and women, the spike-reactive CD8+ T cell response was increased in men. The humoral response is lower in males and elderly individuals, but the way in which sex and age may influence cellular responses to vaccination is barely understood, and the reason for the increase in the spike-reactive CD8+ T cell response with an increase in age in males is unclear [27].

We also found that, after adjusting for other factors, a higher age is associated with a higher anti-S level, which is different than our previous analysis [10,17]. Age is one of the most relevant parameters that determines antibody response; since both T cell-derived antibody production and B lymphocyte generation decrease with age, the antibody response to combat infectious agents following vaccination may not be adequate [28]. Despite that, the last ECDC (European Centre for Disease Prevention and Control) report that investigated SARS-CoV-2 vaccine effectiveness until November 2022 against Severe Acute Respiratory Infection concluded that the relative effectiveness of the booster dose versus the complete primary series suggests a positive relationship with age [29]. Other studies reported this positive correlation with age [30,31,32] and support a stronger humoral response in adults than in younger people. This could be also due to a previous stimulation of the immune system with a cross-reactive coronavirus strain during a longer lifetime [30]. Despite that, a higher age is correlated with worse outcomes from COVID-19 infection [30], opening a debate on the protective role of antibodies. In addition, it should be noted that our study population consisted of mainly healthy, middle-aged workers, and our results might not be directly comparable to those based on patients who are frail or older people. In fact, the higher age considered in our analysis is 65 years.

Immunization determined by various types of vaccines undergoes a progressive decline over time. Several factors influence the immune response to vaccination, including individual factors and intrinsic host factors (like genetics, sex, age and comorbidities), extrinsic factors (such as preexisting immunity, microbiota and antibiotics) and perinatal factors (such as maternal aspects, feeding method and birth weight) [33]. Also, the interval between dose administration has been investigated after the different public authorities in many countries decided to distance vaccine doses in order to enlarge the population coverage with at least one vaccine dose. For the Comirnaty [Pfizer/BioNtech] vaccine, two doses are administered three weeks apart. As reported in some studies, the extension of the interval of the second dose from 3 to 16 weeks in previously infected individuals did not remarkably change the humoral responses, while in never-infected people, this delay elicited a stronger humoral response [34]. Another study supports that delaying the second dose strongly boosts the peak antibody response in older people, while peak cellular-specific responses were higher in those vaccinated respecting the standard 3-week interval [35]. COVID-19 generates a complex humoral and cellular immune response that is still the object of attention in the scientific panorama. The activation of different immune targets, like CD4 T and CD8 T cells and memory B cells, varies considerably and could also be associated with disease severity [36]. According to the literature [18] and to our results [10,17], a progressive decrease in antibody titer is observed over time following the administration of the first dose of vaccine after adjusting for other determinants of the immune response, including the number of doses and vaccine type. Moreover, higher neutralizing antibody titers were detected in vaccinated subjects with previous SARS-CoV-2 infection, not always associated with disease severity [6,24,37], and recent studies observed that IgG antibody titers were higher in subjects with hybrid immunization than in those with no record of natural infection [38].

In this population of HCW, past infection was significantly related to antibody level at 12 months from vaccine administration, consistent with our previous findings. However, the present analysis showed a markedly weaker association than the previous one, which referred to the 9-month serological response [10]. This may reflect a further effect of time on antibody waning.

In this and in previous analyses [10,17,39] within the ORCHESTRA cohorts, we explored the qualitative and quantitative serological responses that correlated with the vaccine characteristics. The huge majority of cohort members were administered with either Comirnaty [BioNTech/Pfizer] or Spikevax [Moderna], which induced an immune response based on mRNA. Our results support several studies that described a longer persistence of antibodies after Spikevax [Moderna] vaccination than Comirnaty [BioNTech/Pfizer] [40] and a higher level of Spikevax [Moderna]-only induced antibodies compared to other vaccines and combinations [41].

To our knowledge, this is the largest cohort of HCW in which the time trends of SARS-CoV-2 vaccine-induced immunization have been studied.

Similar results have been obtained in other countries that administered different vaccines. For example, a Chinese study investigated the immune memory at 1, 3, 6 and 12 months after the two-dose CoronaVac vaccination and found that, after 12 months, the geometric mean titer of antibodies decreased but was still significantly higher than the baseline. However, it is not easy to compare the capacity of different vaccines to produce antibodies due to the different immunization assays used in different laboratories [42].

To prevent the spread of new SARS-CoV-2 variants, an appropriate planning of a vaccine booster is an important task. A large body of literature shows, consistent with our results, how the administration of the third [43,44] and fourth vaccine doses [45] could enhance a recall of memory B cells with a strong production of IgG and neutralizing antibodies compared to two doses in subjects aged over 60 who had a lower response following the second dose [43].

The main strength of the present study lies in its large sample size, including twelve cohorts of HCW from different European centers, and in its prospective design. Our results refer to the effect of a full SARS-CoV-2 vaccine schedule, considering that most of the HCW included in this analysis received a booster dose of vaccine that included the third or fourth dose.

Regarding antibody measurements, the heterogeneity in blood tests (e.g., values obtained from capillary blood samples in the Munich cohort) and in the methods used for antibody detection among the cohorts was addressed by standardizing the results. In this way, we were able to compare the differences in antibody levels among the various cohorts according to several characteristics and to identify the predictors of high immunological response. Our previous studies confirmed the validity of this approach [10,17] and could represent a suitable way to address the issue of heterogeneity in the method of data collection in different populations, as is often the case for large analyses that include multiple centers.

This study aims to provide an update on the serology data of the vaccinated HCW included in the ORCHESTRA pooled study after the results already obtained at 3, 6 and 9 months after vaccination [19]. The present analysis completed the information and evidence collected in previous studies and provides useful epidemiological data on the trend of antibody levels in a large vaccinated European population over a wide time window. Furthermore, the ORCHESTRA project will produce additional results as the follow-up of vaccinated HCW continues, including individual-level trends in antibody levels. Moreover, the robust and well-consolidated statistical methods confer strength to the overall analyses.

A limitation of this study is the scarcity of information for some of our cohorts on health-related factors, such as BMI [46], smoking status and comorbidities, which could have acted as confounder or effect modifiers. Moreover, we did not collect precise information on some characteristics of SARS-CoV-2 infection, like the severity of the symptoms and duration of infection, which could have played an important role in the development of antibodies and their persistence over time [18,35]. Additionally, we did not account for the date of SARS-CoV-2 infection.

Another limitation of this analysis is the use of predicted values of serology in four cohorts in which the serology results were mostly over the cut-off value detectable with the available assay. However, the results of the analysis restricted to the remaining cohorts did not suggest bias from the imputation process. The heterogeneity in the cohorts’ characteristics, primarily the number of participants with available information for the 12-month serology, also represents a limitation, which could still be addressed through the multivariable statistical models by accounting for the study center as a confounder.

These results are consistent with the current literature and support those presented in our previous analyses [10,17]. This analysis focused on different sociodemographic and vaccine-related factors, but additional characteristics may be associated to the development and the persistence of vaccine-induced SARS-CoV-2 immunity, including vaccine-related, host-related (such as genetics and lifestyle), virus-related and environmental [33]. Even if much attention has been dedicated to SARS-CoV-2 infection, these factors remain to be properly explored.

## 5. Conclusions

In conclusion, we provide solid data to support that the antibodies induced by vaccines persist up to 12 months after vaccination with a slight decline over time. These data contribute to the evidence on the serological response to SARS-CoV-2 vaccines and provide information on vaccine-induced immunity persistence and efficacy. These findings can also help to improve vaccination timing and administration of booster doses to better contrast the spreading of the virus, also providing information on the determinants that influence the humoral response The progressive expanding knowledge of SARS-CoV-2 immunization kinetics can help to develop a SARS-CoV-2 vaccination that is more personalized in order to optimize health sources and minimize risks for HCW and the population in general. Only long-term observation from large multicentric prospective studies that cover different populations can provide more definitive and accurate evidence on the predictors of a higher immunity response to vaccination.

## Figures and Tables

**Table 1:** Analytical methods used to measure SARS-CoV-2 antibody level and mean of crude and standardized serology test results by cohort.

Cohort	Assay	Crude Result: Mean (SE) *	Standardized Results: Mean (SE) *
Germany-Munich	Ro-RBD-Ig-quant-DBS#	434.84 (13.85)	4.39 (0.03)
Italy-Bari (IRCCS)	CLIA IgG quantitative	6173.09 (1228.94)	6.85 (0.18)
Italy-Bari (Policlinico)	Abbott SARS-CoV2 IgG II Quant Test	41,379.33 (4459.53)	8.05 (0.12)
Italy-Bologna	Ab anti SARS-CoV-2 S (RBD) IgG ECLIA	2391.58 (14.69)	2.36 (0.03) †
Italy-Brescia	ECLIA Elecsys^®^ anti SARS CoV2 S for anti-SARS-CoV-2-S total antibody detection (Roche Diagnostics International Ltd., Rotkreuz, Switzerland)	4582.78 (12.58)	1.42 (0.01) †
Italy-Padova	LIAISON SARS-CoV-2 TrimericS IgG	7187.21 (139.77)	10.40 (0.02)
Italy-Perugia	Diasorin	1884.96 (21.34)	2.00 (0.05) †
Italy-Trieste	CMIA Abbott anti S-RBD	29,817.64 (1247.10)	5.99 (0.02)
Italy-Verona	DIASORIN LIAISON^®^ SARS-COV-2 TRIMERIC–S–IGG	8515.69 (122.20)	11.90 (0.02)
Romania-Multicenter	Abbot SARS-CoV-2 IgG II Quant test	12,844.52 (910.43)	6.53 (0.07)
Slovakia-Multicenter	Anti-SARS-CoV-2 QuantiVac ELISA (IgG) EUROIMMUN	1330.16 (16.46)	1.45 (0.04) †
Spain-Northern Barcelona region	DECOV1901 ELISA (IgG-S)	3313.32 (76.24)	15.54 (0.05)

* Adjusted by age, according to the Standard European Population; † Cubic transformation; #DBS: dried blood spot from capillary samples, SE: Standard Error. We repeated the analysis separately for the four cohorts with a large number of results extrapolated using the Tobit regression and the remaining eight cohorts. Finally, we compared the cohort-specific crude serology levels at 12 months to those measured at 6 and 9 months [10,17].

**Table 2:** Selected characteristics of subjects included in the analysis.

	Germany-Munich	Italy-Bari (IRCCS)	Italy-Bari (Policlinico)	Italy-Bologna	Italy-Brescia	Italy-Padova	Italy-Perugia	Italy-Trieste	Italy-Verona	Romania-Multicenter	Slovakia-Multicenter	Spain-Northern Barcelona
Total	825	32	66	851	5569	2336	386	2097	2838	217	552	332
Qualitative characteristics (N, (%))												
Sex												
Male	191 (23)	10 (31)	17 (26)	239 (28)	1426 (26)	553 (24)	94 (24)	665 (32)	648 (23)	38 (18)	88 (16)	67 (20)
Female	634 (77)	22 (69)	49 (74)	612 (72)	4143 (74)	1783 (76)	292 (76)	1432 (68)	2190 (77)	179 (82)	464 (84)	265 (80)
Age group												
<=29	133 (16)	17 (53)	13 (20)	84 (10)	773 (14)	188 (8)	4 (1)	196 (9)	359 (13)	10 (5)	58 (11)	24 (7)
30–39	212 (26)	2 (6)	18 (27)	201 (24)	1005 (18)	482 (21)	42 (11)	403 (19)	526 (19)	20 (9)	72 (13)	44 (13)
40–49	170 (21)	4 (13)	14 (21)	186 (22)	1440 (26)	484 (21)	86 (22)	467 (22)	715 (25)	62 (29)	187 (34)	120 ((36)
>=50	309 (38)	9 (28)	21 (32)	380 (45)	2351 (42)	1182 (51)	252 (66)	1031 (49)	1238 (44)	125 (58)	235 (43)	144 (43)
Job title												
Physician	NA	1 (3)	20 (30)	172 (20)	1297 (23)	515 (22)	64 (17)	468 (22)	632 (22)	96 (44)	73 (13)	118 (36)
Technician	NA	4 (12)	4 (6)	102 (12)	483 (9)	155 (7)	62 (16)	311 (15)	293 (10)	59 (27)	38 (7)	NA
Nurse	NA	20 (62)	24 (36)	345 (41)	2042 (37)	1245 (53)	46 (12)	775 (37)	1182 (42)	29 (13)	194 (35)	139 (43)
Administration	NA	NA	3 (5)	40 (5)	681 (12)	80 (3)	24 (6)	124 (6)	214 (8)	24 (11)	61 (11)	45 (14)
Other HCW	NA	7 (22)	15 (23)	191 (22)	1066 (19)	340 (15)	185 (49)	419 (20)	517 (18)	9 (4)	185 (34)	22 (7)
Previous SARS-CoV-2 infection (PCR and/or AntiN)												
Never infected	550 (67)	30 (94)	3 (5)	763 (90)	3657 (66)	2025 (87)	340 (88)	1543 (74)	2347 (83)	168 (77)	258 (47)	112 (34)
Infected at least once	275 (33)	2 (6)	63 (95)	88 (10)	1912 (34)	311 (13)	46 (12)	554 (26)	491 (17)	49 (23)	294 (53)	220 (66)
Type of vaccine												
Only Comirnaty [BioNTech/Pfizer]	494 (60)	32 (100)	66 (100)	821 (96)	4675 (84)	2211 (99.8)	386 (100)	1962 (96)	2838 (100)	208 (96)	529 (96)	107 (32)
Spikevax [Moderna] alone or with other ** vaccines	315 (38)	0 (0)	0 (0)	30 (4)	891 (16)	4 (0.2)	0 (0)	80 (4)	0 (0)	5 (2)	12 (2)	225 (68)
Comirnaty [BioNTech/Pfizer] with other vaccines *** (except Spikevax [Moderna])	16 (2)	0 (0)	0 (0)	0 (0)	3 (0.1)	1 (0.1)	0 (0)	4 (0.2)	0 (0)	4 (2)	11 (2)	NA
Number of vaccine doses												
1 or 2 doses	129 (16)	2 (6)	0 (0)	77 (9)	76 (1)	47 (2)	92 (24)	207 (10)	50 (2)	82 (38)	233 (42)	60 (18)
3 or 4 doses	692 (84)	30 (94)	66 (100)	774 (91)	5493 (99)	2289 (98)	294 (76)	1888 (90)	2788 (98)	135 (62)	319 (58)	272 (82)
Quantitative characteristics												
Standardized quantitative serology at 12 months												
Mean (SD)	4.39 (0.03)	6.85 (0.18)	8.05 (0.12)	2.36 (0.03) *	1.42 (0.01) *	10.40 (0.02)	2.00 (0.05) *	5.99 (0.02)	11.90 (0.02)	6.53 (0.07)	1.45 (0.04) *	15.54 (0.05)
Days between last dose and serology at 12 months												
Range	(1, 388)	(29, 286)	(28, 141)	(1, 517)	(6, 414)	(10, 420)	(29, 470)	(1, 435)	(10, 350)	(9, 392)	(20, 424)	(4, 396)
Mean (SD)	130.95 (3.02)	79.19 (10.82)	82.54 (2.67)	84.97 (3.01)	113.78 (0.41)	78.23 (0.91)	192.99 (5.28)	84.06 (1.88)	49.86 (0.26)	206.49 (8.34)	226.13 (5.58)	106.19 (5.41)
Days between last dose and serology at 12 months (30-day increase) §												
Range	(1, 13)	(1, 10)	(1, 5)	(1, 17)	(1, 14)	(1, 14)	(1, 16)	(1, 15)	(1, 12)	(1, 14)	(1, 15)	(1, 14)
Mean (SD)	4.86 (0.10)	3.12 (0.36)	3.22 (0.10)	3.36 (0.10)	4.30 (0.01)	3.08 (0.03)	6.94 (0.18)	3.29 (0.06)	2.13 (0.01)	7.44 (0.28)	8.04 (0.18)	4.03 (0.18)

*, Cubic transformation; NA, not available. ** other vaccines: Astrazeneca; Johnson & Johnson; Comirnaty [BioNTech/Pfizer] *** other vaccines: Astrazeneca, Johnson & Johnson. SD = standard deviation. §: 30-day increase is a unit, each 30 days of serology measurement after the last dose of vaccine is considered as one unit of increase. Table 3 illustrates the results of the multivariate linear regression on antibody level. In the main analysis, age was directly related to serological response at 12 months with RR = 1.04 (95% CI =1.00–1.08, *p* = 0.04) for a 10-year increase. A negative, non-statistically significant association was observed for male compared to female sex (RR = 0.89, 95% CI = 0.79–1.01). The RR for a 30-day increase in time since last vaccine dose was 0.94 (95% CI = 0.91–0.98). Antibody levels were considerably higher in subjects previously infected compared to those never infected with a slight difference when considering infection detected with PCR or anti-N serology test compared to those detected through only anti-N serology test (RR = 1.78; 95% CI = 1.29–2.45 vs. 1.46, 95% CI = 1.00–1.12). Furthermore, when splitting previous SARS-CoV-2 infection by timing of PCR detection in relation to vaccine administration, a significant increase in serological response was observed in HCW infected after the 1st dose of vaccine (RR 2.80, 95% CI = 1.64–4.77).

**Table 3:** Determinants of standardized antibody levels at 12 months—results of meta-analysis.

Characteristics [Cohorts Included *]	RR	95% CI
Gender ^1^ [all]		
Male	1.00	Ref
Female	0.89	0.79–1.01
Age ^1^ [all]		
10-year increase	1.04	1.00–1.08
Days between last vaccine dose and 12-month serology ^1^ [all]		
30-day increase	0.94	0.91–0.98
Previous SARS-CoV-2 infection (detection: PCR/antiN serology test) ^1^ [all]		
Never infected	1.00	Ref
Infected at least once	1.78	1.29–2.45
Number of doses ¹ [Ge-Mu, It-Bo, It-Br, It-Pa, It-Pe, It-Ts, It-Vr, Ro-Mc, Sk-Mc, Sp-Ba]		
1–2	1.00	Ref
3–4	1.41	0.86–2.32
Job title ¹ [It-Ba(I), It-Ba(II), It-Bo, It-Br, It-Pa, It-Pe, It-Ts, It-Vr, Ro-Mc, Sk-Mc, Sp-Ba]		
Physician, including resident	1.00	Ref
Nurse	1.05	0.96–1.13
Technician	1.08	0.97–1.19
Administration	1.09	1.02–1.16
Other, including auxiliary workers	1.01	0.94–1.08
Type of vaccine [Ge-Mu, It-Bo, It-Br, It-Pa, It-Ts, Ro-Mc, Sk-Mc, Sp-Ba]		
Only Comirnaty [Pfizer/BioNTech]	1.00	Ref
Spikevax [Moderna] alone or with other vaccines	1.07	0.97–1.19
Comirnaty [Pfizer/BioNTech] with other vaccines (except Spikevax [Moderna])	0.77	0.60–0.98
Previous SARS-CoV-2 infection (detection: PCR) [all]		
Never infected	1.00	Ref
Infected before vaccination	1.35	0.98–1.85
Infected after 1st dose of vaccine	2.80	1.64–4.77
Infected at both times	1.82	0.87–3.77
Previous SARS-CoV-2 infection (detection: antiN serology test) ** [ Ge-Mu, It-Br, Sp-Ba]		
Never infected	1.00	Ref
Infected at least once	1.46	1.00–2.12

RR, relative risk for one SD increase in standardized antibody level, adjusted by age, gender, job title, previous SARS-CoV-2 infection, number of doses, type of vaccine and days between last dose and serology at 12 months; CI, confidence interval; Ref, reference category; ^1^, Adjusted by age, gender, job title, previous SARS-CoV-2 infection, number of doses, type of vaccine and days between last dose and serology at 12 months, as appropriate; * Ge-Mu, Germany-Munich; It-Ba(I), Italy-Bari(IRCCS); It-Ba(II), Italy-Bari(Policlinic); It-Bo, Italy-Bologna; It-Br, Italy-Brescia; It-Pa, Italy-Padova; It-Pe, Italy-Perugia; It-Ts, Italy-Trieste; It-Vr, Italy-Verona; Ro-Mc, Romania-Multicenter; Sk-Mc, Slovakia-Multicenter; Sp-Ba, Spain-Barcelona; ** available for 6661 subjects. Note: Germany-Munich cohort is excluded from the analyses of job title.

## Data Availability

The datasets generated during the current study can be made available in de-identified format upon reasonable request to the principal investigators of the participating cohorts.

## Supplementary Materials

The following supporting information can be downloaded at: https://www.mdpi.com/article/10.3390/vaccines11101527/s1, Table S1: Determinants of standardized antibody level at 12-month, by method of standardization.
