# Supplementary material for: Determinants of Anti-S Immune Response at 12 Months after SARS-CoV-2 Vaccination in a Multicentric European Cohort of Healthcare Workers—ORCHESTRA Project
Source: Vaccines (Basel). 2023 Sep 26;11(10):1527. doi: 10.3390/vaccines11101527 (PMC10610704; doi:10.3390/vaccines11101527)
Supplement: Supplementary file 1 [file vaccines-11-01527-s001.zip › vaccines-2566607-supplementary.pdf]

Supplementary Table S1. Determinants of standardized antibody level at 12-month, by method of standardization

**Characteristics‡**	**Log-10 stand.*<br>RR (95% CI)**	**Cubic stand.†<br>RR (95% CI)**
**Gender**		
Male	1.00 Ref	1.00 Ref
Female	0.94 (0.86-1.26)	0.81 (0.59-1.12)
**Age**		
10-year increase	1.07 (1.03-1.11)	0.99 (0.95-1.04)
**Days between last vaccine dose and 12-month serology**		
30-day increase	0.92 (0.87-0.97)	0.98 (0.95-1.01)
**Previous SARS-CoV-2 infection (detection: PCR/antiN serology test)**		
Never infected	1.00 Ref	1.00 Ref
Infected at least once	1.50 (1.19-1.88)	2.54 (1.66-3.88)
**Number of doses**		
1-2	1.00 Ref	1.00 Ref
3-4	1.12 (0.84-1.49)	2.11 (0.57-7.83)
**Job title**		
Physician, including resident	1.00 Ref	1.00 Ref
Nurse	1.15 (0.95-1.38)	1.03 (0.94-1.12)
Technician	1.06 (0.93-1.22)	1.03 (0.92-1.15)
Administration	1.09 (0.94-1.27)	1.07 (1.01-1.14)
Other, including auxiliary workers	1.04 (0.91-1.19)	0.98 (0.93-1.04)
**Type of vaccine**		
Only Comirnaty	1.00 Ref	1.00 Ref
Spikevax alone or with other vaccines	1.02 (0.88-1.17)	1.17 (1.10-1.24)
Comirnaty with other vaccines (except Spikevax)	0.83 (0.55-1.25)	0.69 (0.45-1.04)
**Previous SARS-CoV-2 infection (detection: PCR)**		
Never infected	1.00 Ref	1.00 Ref
Infected before vaccination	0.99 (0.84, 1.18)	2.75 (0.92, 8.18)
Infected after 1st dose of vaccine	2.43 (1.88, 3.15)	3.60 (1.62, 7.97)
Infected at both times	1.04 (0.75, 1.43)	5.74 (2.59, 12.72)
**Previous SARS-CoV-2 infection (detection: antiN serology test)**		
Never infected	1.00 Ref	1.00 Ref
Infected at least once	1.62 (1.01, 2.60) **	1.20 (1.13, 1.28) ††

RR, relative risk for one SD increase in standardized antibody level, adjusted by age, gender, job title, previous SARS-CoV-2 infection, number of doses, type of vaccine, and days between last dose and serology at 12-month.

CI, confidence interval

Ref, reference category

1, adjusted by age, gender, job title, previous SARS-CoV-2 infection, number of doses, type of vaccine, and days between last dose and serology at 12-month, as appropriate

* Germany-Munich; Italy-Bari; Italy-Padova; Italy-Trieste; Italy-Verona; Romania-Multicenter; Spain-Northern Barcelona

** available for Germany-Munich and Spain-Northern Barcelona only

† Italy-Bologna; Italy-Brescia; Italy-Perugia; Slovakia-Multicenter

†† available for Italy-Brescia only.

‡ See Table 3 for availability of data in each cohort
